# Hyaluronic acid turnover controls the severity of cerebral cavernous malformations in bioengineered human micro-vessels

**DOI:** 10.1063/5.0159330

**Published:** 2024-02-12

**Authors:** Teodor E. Yordanov, Mikaela S. Keyser, Marco A. Enriquez Martinez, Tyron Esposito, Juliann B. Tefft, Elysse K. Morris, Larisa I. Labzin, Samantha J. Stehbens, Alan E. Rowan, Benjamin M. Hogan, Christopher S. Chen, Jan Lauko, Anne K. Lagendijk

**Affiliations:** 1 Centre for Cell Biology and Chronic Disease, Institute for Molecular Bioscience, The University of Queensland, Brisbane, Queensland, Australia; 2 Australian Institute for Bioengineering and Nanotechnology, The University of Queensland, Brisbane, Queensland, Australia; 3 Centre for Inflammation and Disease Research, Institute for Molecular Bioscience, The University of Queensland, Brisbane, Queensland, Australia; 4 The Biological Design Center and Department of Biomedical Engineering, Boston University, Boston, Massachusetts, 02215, USA; 5 School of Biomedical Sciences, Faculty of Medicine, The University of Queensland, Brisbane, Queensland, Australia; 6 Organogenesis and Cancer Program, Peter MacCallum Cancer Centre, Melbourne, Victoria, Australia; 7 Department of Anatomy and Physiology, University of Melbourne, Melbourne, Victoria, 02215, Australia; 8 The Wyss Institute for Biologically Inspired Engineering, Harvard University, Boston, Massachusetts, 02215, USA

## Abstract

Cerebral cavernous malformations (CCMs) are vascular lesions that predominantly form in blood vessels of the central nervous system upon loss of the CCM multimeric protein complex. The endothelial cells within CCM lesions are characterized by overactive MEKK3 kinase and KLF2/4 transcription factor signaling, leading to pathological changes such as increased endothelial cell spreading and reduced junctional integrity. Concomitant to aberrant endothelial cell signaling, non-autonomous signals from the extracellular matrix (ECM) have also been implicated in CCM lesion growth and these factors might explain why CCM lesions mainly develop in the central nervous system. Here, we adapted a three-dimensional microfluidic system to examine CCM1 deficient human micro-vessels in distinctive extracellular matrices. We validate that pathological hallmarks are maintained in this model. We further show that key genes responsible for homeostasis of hyaluronic acid, a major extracellular matrix component of the central nervous system, are dysregulated in CCM. Supplementing the matrix in our model with distinct forms of hyaluronic acid inhibits pathological cell spreading and rescues barrier function. Hyaluronic acid acts by dampening cell–matrix adhesion signaling in CCM, either downstream or in parallel of KLF2/4. This study provides a proof-of-principle that ECM embedded 3D microfluidic models are ideally suited to identify how changes in ECM structure and signaling impact vascular malformations.

## INTRODUCTION

Cerebral cavernous malformations (CCMs) are raspberry shaped vascular lesions that manifest primarily in postcapillary venules of the central nervous system (CNS). Lesions can develop sporadically or be a consequence of familial mutations. Both sporadic and familial cases are associated with loss of function (LOF) mutations in vascular endothelial cells (ECs) for one of three genes: *KRIT1* (*CCM1*), *OSM* (*CCM2*), or *PDCD10* (*CCM3*).^1,2^ CCM patients can develop a variety of symptoms depending on the number, size, and location of lesions. Symptoms include headaches, neurological deficits such as seizures and epilepsy, and hemorrhagic stroke.^3–5^

CCM proteins can assemble as a multimeric complex^6,7^ that regulates the activity of an endothelial specific Mitogen-Activated Protein Kinase (MAPK)-MEKK3 pathway.^8–12^ This ectopic MEKK/ERK signaling in CCM lesions leads to excessive KLF2 and 4 transcription factor activity, which has been proven to be a key driver of the disease.^13–16^ Once this pathway has been initiated, CCM lesion growth occurs by clonal expansion and incorporation of neighboring wild-type ECs.^17,18^ Wildtype incorporation is suggested to occur via non-cell autonomous factors. Indeed loss of CCM alters the ultrastructure of the extracellular matrix (ECM) microenvironment,^19–21^ and excessive degradation of the ECM proteoglycan Versican by Adamts5^16^ has been shown to directly contribute to CCM lesion growth.^22^ CCM1 can also dampen the activation of the cell–matrix adhesion receptor β1-integrin by stabilizing the integrin inhibitor ICAP1.^23,24^ Loss of CCM1, therefore, leads to enhanced integrin signaling, which has been shown to contribute to the pathological increase in endothelial cell size.^23,24^ Despite the appreciation that non-cell autonomous factors play an important role in CCM disease progression, it remains challenging to specifically modify and study such ECM components *in vivo*.

To facilitate analysis of ECM structure and signaling in CCM pathogenesis, we here adapted a three-dimensional (3D) microfluidic system^25,26^ to culture human CCM1 deficient micro-vessels. These vessels are grown under flow and in tunable ECM hydrogels. We applied this model to grow CCM vessels in brain-mimetic matrices, enriched with hyaluronic acid (HA), the major structural component of the brain ECM.^27,28^ HA molecules can vary greatly in size, ranging from very short HA oligomers (oHA) up to long polymers, referred to as high molecular weight (HMW) HA, of several MDa.^29–31^ Notably, HA synthesizing and degrading genes are amongst the most dysregulated genes in CCM lesions.^32–34^ We validated that such transcriptional changes are also apparent in our model. We hypothesize that these changes in HA homeostasis would result in a reduced presence of low molecular weight (LMW) HA and excessive turnover of HMW HA in the ECM surrounding CCM lesions. Since distinctly sized HA molecules are appreciated to activate differential cellular signaling,^29–31^ altered HA composition of the ECM would likely impact CCM pathology. We used our CCM micro-vessel model to test this by monitoring changes in endothelial cell phenotypes when CCM micro-vessels are grown in distinct HA environments. We identified that LMW HA prevents pathological EC enlargement. Composite ECMs containing both HMW and LMW HA created the most favorable environment, rescuing EC size and junctional gap formation. Analysis of focal adhesion formation suggest that the protective role of LMW HA molecules acts at the level of integrin signaling. These significant improvements of CCM cellular phenotypes imply that restoration of HA homeostasis might prevent CCM progression and inhibit the formation of unstable and leaky lesions.

## RESULTS AND DISCUSSION

### Establishing a human CCM micro-vessels model to study ECM interactions

**FIG. 1.**
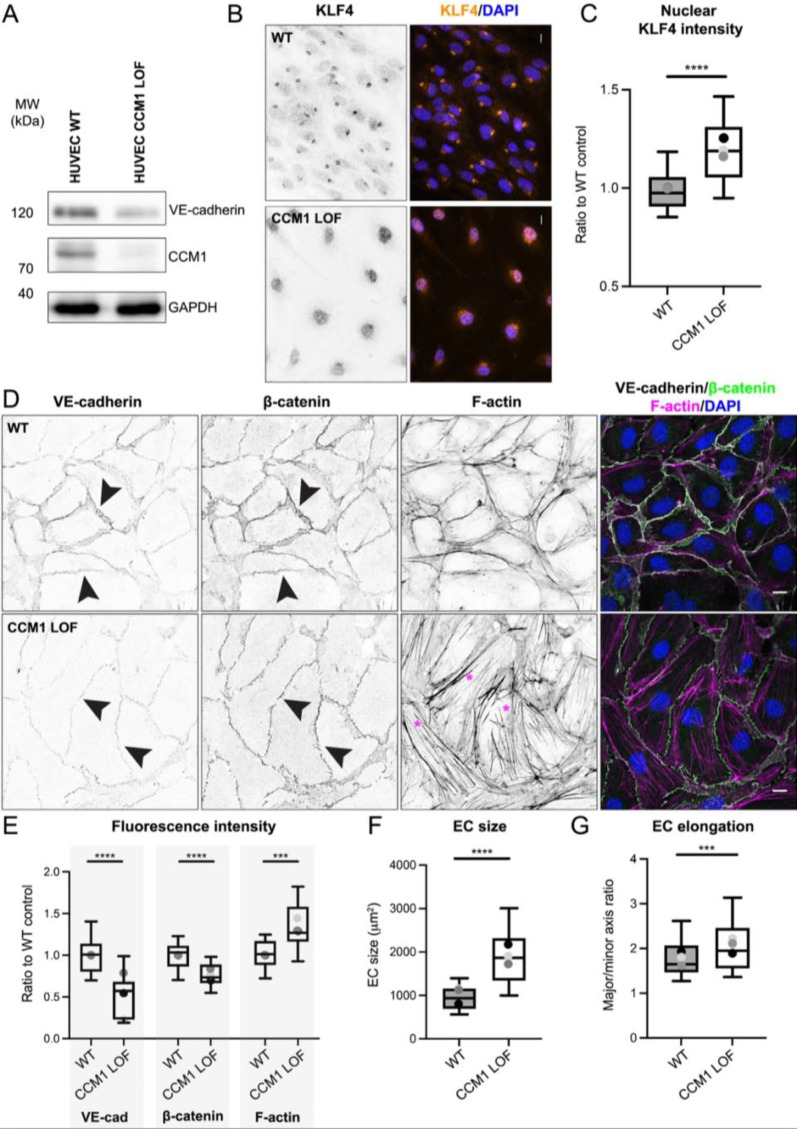
Generation and characterization of CCM1 LOF HUVECs. (a) Western blot of WT and CCM1 LOF HUVECs showing loss of CCM1 (KRIT-1) protein expression. (b) Immunofluorescence of WT and CCM1 LOF ECs, stained with KLF4 and DAPI. Scale bar: 10 *μ*m. Left panel: KLF only (gray). Right panel: KLF4 (orange) and DAPI (blue). (c) Box and whisker plot of KLF4 fluorescence intensity in the EC nuclei. Mean value of each independent replicate is represented as a dot with matching colors between WT and CCM1 LOF. n = 3 replicates; n = 150 WT and n = 138 CCM1 LOF ECs, Mann–Whitney test ^****^p < 0.0001. (d) Immunofluorescence of WT and CCM1 LOF ECs, stained for VE-cadherin, β-catenin, Phalloidin (F-actin) and DAPI. Arrowheads show adherens junctions, marked by VE-cadherin and β-catenin and asterisks (magenta) indicate F-actin stress fibers in CCM1 LOF ECs. Scale bar 10 *μ*m. (e) Box and whisker plot indicating fluorescence intensity of VE-cadherin, β-catenin, and Phalloidin (F-actin). Mean value of each replicate is represented as a dot with matching colors between WT and CCM1 LOF. n = 3 replicates; n = 18 WT and n = 18 CCM1 LOF ECs, Student's t-test ^****^p < 0.0001 and ^***^p < 0.001. (f) Quantification of the cell size of WT and CCM1 LOF ECs, based on VE-cadherin staining. Box and whisker plot with the mean value of each replicate represented as a dot with matching colors between WT and CCM1 LOF ECs. n = 3 replicates; n = 150 WT and n = 138 CCM1 LOF ECs, Mann–Whitney test ^****^p < 0.0001. (g) Quantification of cell elongation (major axis over minor axis) of WT and CCM1 LOF ECs, utilizing VE-cadherin to demarcate the cell boundaries. Box and whisker plot with mean value of each replicate represented as a dot with matching colors between WT and CCM1 LOF ECs. n = 3 replicates; n = 150 WT; and n = 138 CCM1 LOF ECs, Mann–Whitney test ^****^p < 0.0001.

For this study we aimed to interrogate how changes in the ECM might alter the severity of EC phenotypes within CCM vasculature. First, we generated a CCM1/KRIT1 loss of function (LOF) model in human umbilical cord endothelial cells (HUVECs) using CRISPR/Cas9, further referred to as CCM1 LOF [Fig. 1(a)]. Growing these CCM1 LOF cells in a 2D setting revealed increased KLF4 expression in the EC nuclei [Figs. 1(b) and 1(c)], validating CCM pathological signaling was activated.^13,15^ CCM1 LOF ECs also displayed thinner cell–cell junctions with significantly reduced expression of the adheren junction proteins such as vascular endothelial (VE)-cadherin and β-catenin [Figs. 1(d) and 1(e)]. By utilizing VE-cadherin expression at the junctions, we further determined that CCM1 LOF ECs were larger and more elongated than their wild-type (WT) counterparts [Figs. 1(d), 1(f), and 1(g)]. In agreement with previous studies,^15,35,36^ CCM1 LOF ECs contained prominent (F)-actin rich stress fibers [Figs. 1(d) and 1(e)] with elevated levels of phosphorylated Myosin Light Chain (pMLC S19 and pMLC T18/S19) (supplementary material Fig. 1), confirming increased actomyosin contractility.

**FIG. 2.**
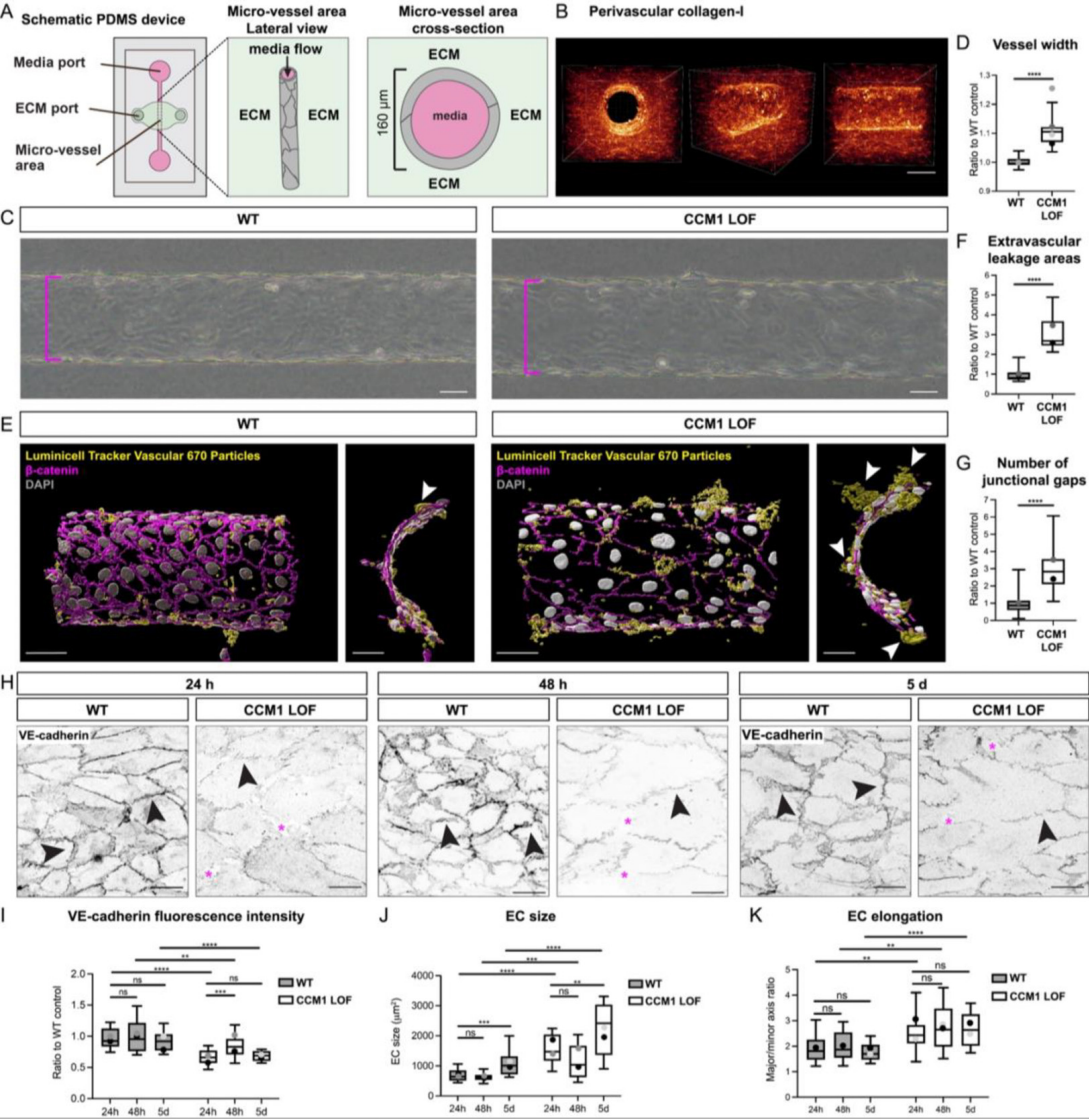
Establishing a human model of CCM deficient 3D vasculature. (a) Schematic of microfluidic device in which the 3D micro-vessel is generated. (b) Reflection imaging of perivascular collagen-I in extracellular matrix surrounding micro-vessels (scale bar = 60 *μ*m). (c) Brightfield images of WT and CCM1 LOF micro-vessels grown in 2.5 mg/ml collagen-I ECMs (scale bar = 50 *μ*m). Magenta brackets indicate vessel diameter size, which is increased in CCM1 LOF micro-vessel. (d) Vessel diameter (width) quantification, demonstrating an increase in vessel diameter in CCM1 LOF vessels; n = 4 replicates; n = 20 WT and n = 20 CCM1 LOF micro-vessels, Student's t-test ^****^p < 0.0001. (e) 3D surface rendering of immunofluorescent staining for cell–cell junctions (β-catenin, magenta) and nuclei (DAPI, gray), and Luminicell Tracker Vascular 670 nanoparticles (yellow). Vascular leakage of Luminicell Tracker Vascular 670 particles is increased in CCM1 LOF micro-vessels (scale bar = 50 *μ*m). Leakage points are indicated by white arrowheads. (f) Quantification of extravascular leakage areas, based on extravascular presence of Luminicell Tracker Vascular 670 particles; n = 2 replicates, n = 11 WT and n = 11 CCM1 LOF micro-vessels, Student's t-test ^****^p < 0.0001. (g) Quantification of WT and CCM1 LOF junctional gaps. Box and whisker plot with mean value of each replicate represented as a dot with matching colors between WT and CCM1 LOF ECs. n = 2 replicates; n = 37 WT and n = 37 CCM1 LOF ROIs, collagen-I and 0.1% 500–749 kDa HA and HAse n = 33 WT and n = 29 CCM1 LOF ROIs, collagen-I and 0.1% <10 kDa HA, 0.1% 41–65 kDa HA and 0.1% 500–749 kDa HA n = 36 WT and n = 34 CCM1 LOF ROIs, Student's t-test ^****^p < 0.0001. (h) Immunofluorescence of WT and CCM1 LOF EC cells, seeded in 3D microfluidic devices, grown for 24 h (left), 48 h (middle), and 5 d (right) post seeding, and stained for VE-cadherin (gray), Scale bar: 25 *μ*m. Arrowheads indicate VE-cadherin at cell–cell junctions and asterisks indicate junctional gaps. (i) Quantification of the VE-cadherin fluorescence intensity in ECs of WT and CCM1 LOF micro-vessels. Box and whisker plot with mean value of each replicate represented as a dot with matching colors between WT and CCM1 LOF ECs. n = 3 replicates; 24 h: n = 39 WT and n = 28 CCM1 LOF ECs, 48 h: n = 33 WT and n = 27 CCM1 LOF ECs, 5 d: n = 24 WT and n = 19 CCM1 LOF ECs, Student's t-test ^****^p < 0.0001, ^***^p < 0.001, ^**^p < 0.005, ns = no significant difference. (j) Quantification of EC size in WT and CCM1 LOF micro-vessels. Box and whisker plot with mean value of each replicate represented as a dot with matching colors between WT and CCM1 LOF ECs. n = 3 replicates; 24 h n = 28 WT and n = 22 CCM1 LOF ECs, 48 h n = 33 WT and n = 27 CCM1 LOF ECs, 5 d n = 24 WT and n = 17 CCM1 LOF ECs, Mann Whitney test ^****^p < 0.0001, ^***^p < 0.001, ^**^p < 0.005, ns = no significant difference. (k) Quantification of EC elongation (major over minor axis) of ECs in WT and CCM1 LOF micro-vessels. Box and whisker plot with mean value of each replicate represented as a dot with matching colors between WT and CCM1 LOF ECs. n = 3 replicates; 24 h n = 28 WT and n = 22 CCM1 LOF ECs, 48 h n = 33 WT and n = 27 CCM1 LOF ECs, 5 d n = 24 WT and n = 17 CCM1 LOF ECs, Student's test ^****^p < 0.0001, ^**^p < 0.005, ns = no significant difference.

Next, we adapted a bio-engineered 3D model that was established by Polacheck and colleagues^25,26^ to grow CCM LOF micro-vessels [Fig. 2(a)]. In this setup, the ECs are seeded on the luminal side of a 3D tube, surrounded by a physiologically relevant ECM [Fig. 2(b)], and placed under constant laminar flow pressure, thereby modeling the geometrical and environmental cues found *in vivo*. This micro-vessel model is particularly suited to advance studies into the role of ECM in CCM pathogenesis since it allows for quantitative analysis of cellular phenotypes as well as overall vessel morphology in distinct matrices, while keeping other parameters constant.

We next seeded our validated CCM1 LOF ECs into a 3D collagen-I ECM (2.5 mg/ml), the standardized ECM hydrogel used in this model.^25,26^ Following the attachment of the ECs, the vessels were placed on a rocking platform to establish gravity-driven oscillatory flow.^25,26^ We analyzed overall vessel morphology and barrier function of the micro-vessels. At 48 h post seeding, CCM1 LOF micro-vessels were larger, demonstrated by a significant increase in vessel diameter [Figs. 2(c) and 2(d)], a well-known feature of CCM deficient vasculature. To determine whether vessel expansion was accompanied by compromised barrier function, we added fluorescent nanoparticles into the lumen of these micro-vessels. We identified a significant increase in leakage areas, where nanoparticles were present in the extravascular space [Figs. 2(e) and 2(f)]. This reduction in barrier function was further explained by an increase in the number of junctional gaps in CCM1 LOF micro-vessels [Fig. 2(g)].

At the cellular level, we validated that CCM cellular characteristics were present in this 3D configuration as early as 24 h post EC seeding, and that these phenotypes were maintained for at least five days. We observed a decrease in VE-cadherin coverage [Figs. 2(h) and 2(i)], an increase in EC size [Figs. 2(h) and 2(j)], and enhanced EC elongation [Figs. 2(h) and 2(k)]. Together these experiments have validated that CCM1 LOF micro-vessels are a suitable model to study CCM biology.

**FIG. 3.**
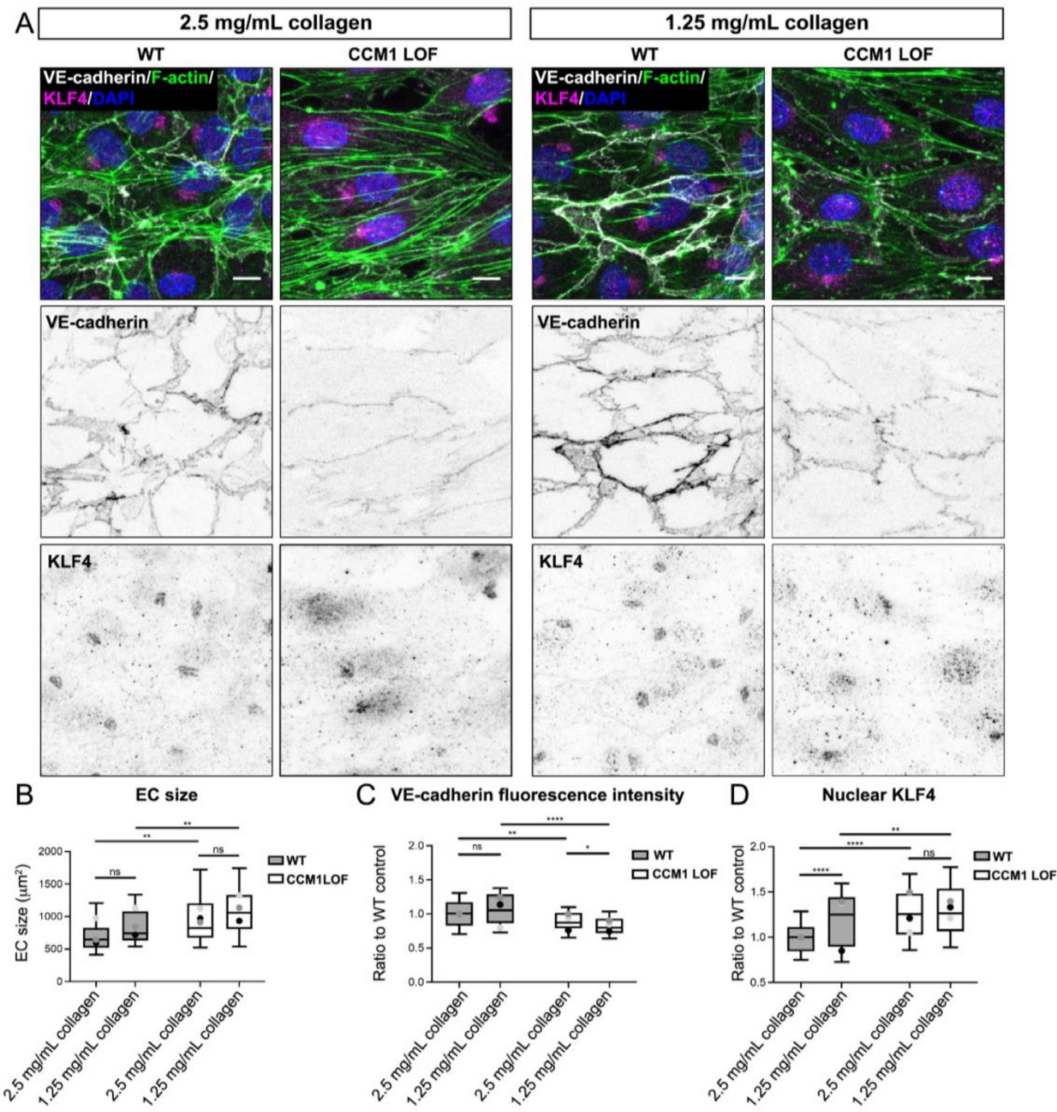
Decreasing collagen content and stiffness does not impact CCM severity. (a) Immunofluorescence of ECs in WT and CCM1 LOF micro-vessels grown for 48 h in either 2.5 mg/ml collagen-I (left) or 1.25 mg/ml collagen-I (right), and stained for VE-cadherin, Phalloidin (F-actin), KLF4 and DAPI. Scale bar: 10 *μ*m. (b) Quantification of cell size of ECs in WT and CCM1 LOF micro-vessels grown in either 2.5 or 1.25 mg/ml collagen-I. Box and whisker plot with mean value of each replicate represented as a dot with matching colors between WT and CCM1 LOF ECs. n = 3 replicates; 2.5 mg/ml collagen-I n = 58 WT and n = 49 CCM1 LOF ECs, 1.25 mg/ml collagen-I n = 51 WT and n = 54 CCM1 LOF ECs, Mann Whitney test ^**^p < 0.005, ns = no significant difference. (c) Quantification of the VE-cadherin fluorescence intensity in ECs of WT and CCM1 LOF micro-vessels grown in either 2.5 or 1.25 mg/ml collagen-I. Box and whisker plot with mean value of each replicate represented as a dot with matching colors between WT and CCM1 LOF ECs. n = 3 replicates; 2.5 mg/ml collagen-I n = 58 WT and n = 49 CCM1 LOF ECs, 1.25 mg/ml collagen-I n = 51 WT and n = 54 CCM1 LOF ECs, Student's t-test ^***^p < 0.001, ^**^p < 0.005, ^*^p < 0.05, ns = no significant difference. (d) Quantification of the amount of nuclear KLF4, based on overlapping signal from KLF4 and DAPI. Box and whisker plot with mean value of each replicate represented as a dot with matching colors between WT and CCM1 LOF ECs. n = 3 replicates; 2.5 mg/ml collagen-I n = 159 WT and n = 164 CCM1 LOF ECs, 1.25 mg/ml collagen-I n = 165 WT and n = 132 CCM1 LOF ECs, Mann–Whitney test ^****^p < 0.0001, ^**^p < 0.005, ^*^p < 0.05, ns = no significant difference.

Since CCM lesions form predominantly in the CNS, where the ECM is known to be much softer compared to many other tissues,^37,38^ we utilized our 3D model to determine whether an extremely soft environment would enhance CCM cellular phenotypes. We reduced the collagen-I concentration in the 3D hydrogel from 2.5 to 1.25 mg/ml. This resulted in a fivefold reduction of the ECM stiffness, from ∼400 to 80 Pa, whilst maintaining the collagen structure and pore size [(supplementary material Figs. 2(a)–2(c)]. Micro-vessels were grown in these ECMs for 48 h post seeding and subsequently fixed for cellular analysis. Quantifications of EC size [Figs. 3(a) and 3(b)], junctional VE-cadherin intensity [Figs. 3(a) and 3(c)] and nuclear KLF4 intensity [Figs. 3(a) and 3(d)], uncovered that in CCM1 ECs exposed to 1.25 mg/ml collagen VE-cadherin intensity was reduced [Figs. 3(a) and 3(c)]. All other CCM pathological phenotypes were unchanged. These data indicate that growing CCM vessels in a highly compliant ECM does not significantly alter phenotypic outcome, suggesting that low tissue stiffness in the CNS is not sufficient to induce CCM formation.

### CCM1 loss alters transcriptional profiles of HA metabolism genes

**FIG. 4.**
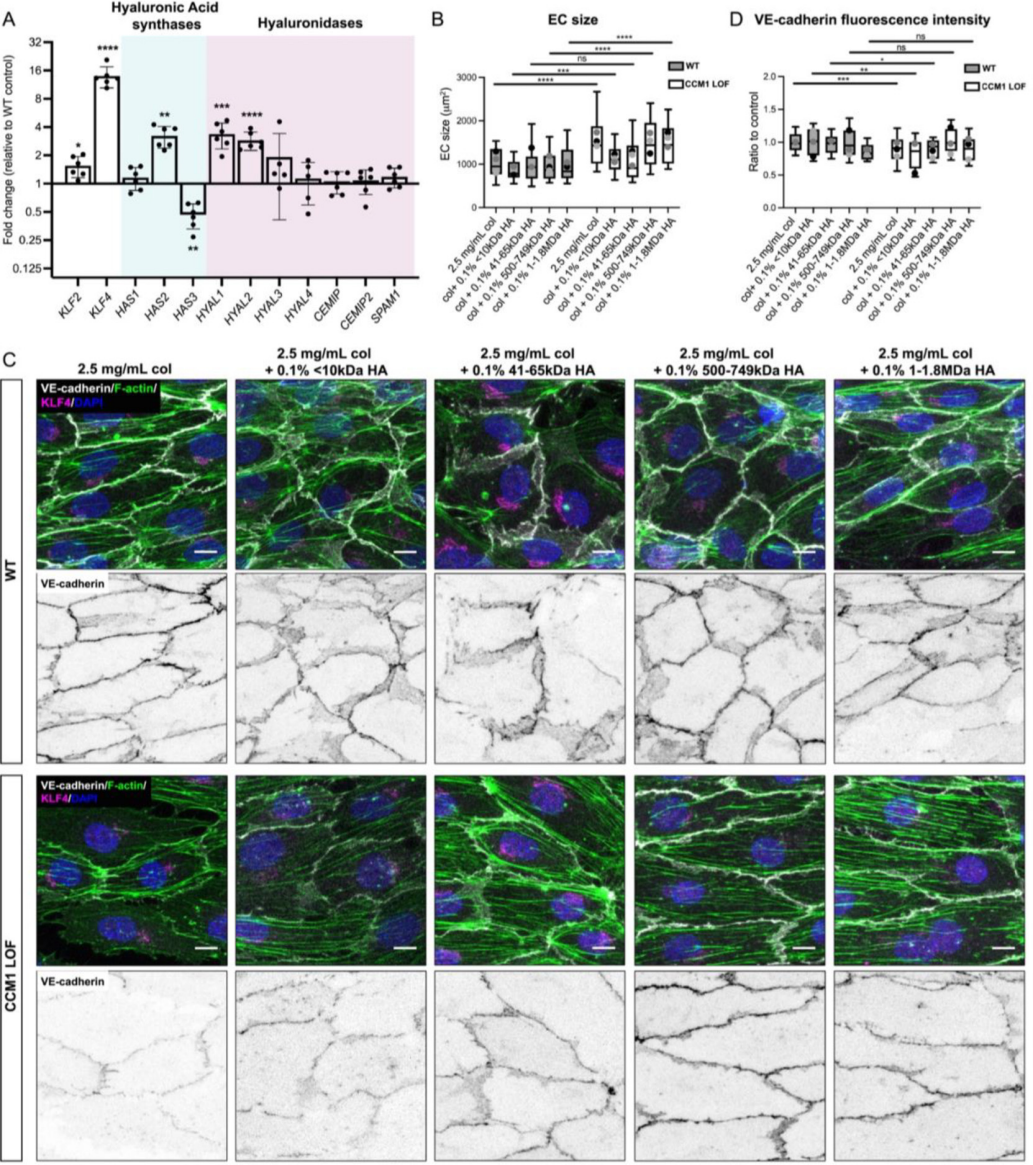
Impact of HA of defined molecular weights on CCM phenotype. (a) Quantitative RT-PCR analysis on mRNA isolated from WT and CCM1 LOF EC cells. Data presented as relative fold change to WT. Cyan background = hyaluronic acid synthases and magenta background = hyaluronidases. Expression of each gene was corrected relative to changes in the housekeeping gene *Hypoxanthine Phosphoribosyltransferase 1* (*HPRT*). Every dot-point represents a technical replicate (total of n = 3 technical replicates) from n = 2 independent biological replicates. Bars represent mean value. Error bars represent the standard deviation. (b) Quantification of the cell size of WT and CCM1 LOF EC cells, measured based on VE-cadherin staining. Box and whisker plot with mean value of each replicate represented as a dot with matching colors between WT and CCM1 LOF ECs. n = 3 replicates; 2.5 mg/ml collagen-I n = 66 WT and n = 52 CCM1 LOF ECs, collagen-I and 0.1% <10 kDa HA n = 41 WT and n = 36 CCM1 LOF ECs, collagen-I and 0.1% 41–65 kDa HA n = 45 WT and n = 50 CCM1 LOF ECs, collagen-I and 0.1% 500–749 kDa HA n = 44 WT and n = 42 CCM1 LOF ECs, collagen-I and 0.1% 1–1.8 MDa HA n = 44 WT and n = 38 CCM1 LOF ECs. Mann–Whitney test ^****^p < 0.0001, ^***^p < 0.001, ns = no significant difference. (c) Immunofluorescence of WT and CCM1 LOF EC cells, seeded in 3D microfluidic devices in ECMs composed of either collagen-I only, or collagen-I with 0.1% of the hyaluronic acid (HA) of incremental molecular weights. Tubes were grown for 48 h post seeding and stained for VE-cadherin (white), Phalloidin (F-actin) (green), KLF4 (magenta) and DAPI (blue). Scale bar: 10 *μ*m. (d) Quantification of VE-cadherin fluorescence intensity at cell–cell junctions in WT and CCM1 LOF ECs. Box and whisker plot with mean value of each replicate represented as a dot with matching colors between WT and CCM1 LOF ECs. n = 3 replicates; 2.5 mg/ml collagen-I n = 133 WT and n = 107 CCM1 LOF ECs, collagen-I and 0.1% <10 kDa HA n = 41 WT and n = 36 CCM1 LOF ECs, collagen-I and 0.1% 41–65 kDa HA n = 45 WT and n = 50 CCM1 LOF ECs, collagen-I and 0.1% 500–749 kDa HA n = 43 WT and n = 41 CCM1 LOF ECs, collagen-I and 0.1% 1–1.8 MDa HA n = 41 WT and n = 42 CCM1 LOF ECs. Mann–Whitney test ^***^p < 0.001, ^**^p < 0.005, ^*^p < 0.05, ns = no significant difference.

We next interrogated whether ECM composition can impact CCM severity. The main ECM constituent of the brain is the glycosaminoglycan Hyaluronic acid (HA), which underpins unique CNS properties.^27,39^ HA is a particularly interesting component of the ECM as HA can elicit distinct biological responses, based on the length of the polysaccharide.^29–31^ Disruption of HA homeostasis has been associated with a wide range of pathologies, especially in the context of inflammation.^40–43^ In the vasculature, distinct forms of HA have been identified to be required for developmental neo-angiogenesis^44^ and to control EC integrity.^45^ HA production and turnover, therefore, needs to be tightly controlled. This occurs through the combined action of HA synthases (HAS) and Hyaluronidases. Notably, *HAS* genes and Hyaluronidases are among the most dysregulated genes in CCM1 deficient lesions from mice.^32^ To determine the scope of CCM1 induced changes on HA metabolism genes, we performed quantitative mRNA expression analysis of all known HA synthases and Hyaluronidases in CCM1 LOF, and WT ECs [Fig. 4(a)]. CCM1 LOF ECs exhibited a marked increase in *HAS2* expression, whereas *HAS3* was significantly down regulated. HAS2 is associated with the synthesis of high-molecular-weight (HMW) HA (>500 kDa), whereas HAS3 produces low-molecular-weight (LMW) HA (<500 kDa).^30,46,47^ Furthermore, Hyaluronidases *HYAL1* and *HYAL2* were significantly upregulated in CCM1 LOF ECs. Since HYAL1 and HYAL2 digest HMW HA into HA polymers of approximately 20 kDa,^30^ this combined expression data implies that LMW HA production is decreased and that HMW HA is present, yet excessively degraded into HA polymers by HYAL1 and HYAL2. Interestingly, such HA polymers have been reported to have a pro-inflammatory effect via combined TLR4 and CD44 receptor activity.^48^ Notably, TLR4 activation has been identified as a pre-requisite of CCM formation.^49^ Changes in HA homeostasis in the CCM microenvironment, therefore, could alter signaling capabilities and impact the progression of this disease.

### HA of distinct molecular sizes can alter CCM cellular phenotypes

Based on the knowledge that HA can have strong effects EC biology, we used our 3D CCM model to test the phenotypic consequences when growing CCM vessels in matrices of predefined HA content. We first examined the impact of HA of distinct molecular weights, including short HA oligomers (<10 kDa), LMW HA (41–65 kDa), and HMW HA of 500–749 kDa and 1–1.8 MDa. We added 0.1% of each HA type to the standard 2.5 mg/ml collagen-I ECM, allowing HA to interconnect with the collagen matrix.^50–52^ The resulting ECMs were similar in terms of stiffness, collagen fiber architecture, and collagen pore size [supplementary material Figs. 2(d)–2(f)]. CCM1 LOF and WT micro-vessels were grown in these distinct ECMs for 48 h and subsequently fixed for phenotypic analysis. By utilizing VE-cadherin expression at the cell–cell junctions, we quantified EC size and identified that addition of LMW HA (41–65 kDa) significantly reduced the size of CCM1 LOF ECs, making them indistinguishable from WT ECs [Figs. 4(b) and 4(c)]. Notably, KLF4 was still induced across all HA conditions [supplementary material Figs. 3(a) and 3(b)]. These data suggest that LMW HA can govern EC size by acting either downstream or in parallel to KLF4. Increasing the concentration of LMW HA in the ECM resulted in the same EC size rescue [supplementary material Figs. 4(a) and 4(b)], suggesting EC size recovery is not HA concentration dependent. Degradation of LMW HA in the ECM using a Hyaluronidase (HAse) from *Streptomyces hyalurolyticus* abolished EC size rescue, validating LMW HA is required to control EC size [supplementary material Figs. 4(a) and 4(b)]. This reversal was not apparent when adding HAse to 1% 41–65 kDa HA ECMs [supplementary material Figs. 4(a) and 4(b)], suggesting that this amount of LMW HA could not be fully degraded by HAse and that sufficient LMW HA molecules remained to inhibit cellular expansion. As observed previously (supplementary material Fig. 3), nuclear intensity of KLF4 across these LMW HA conditions remained unchanged [supplementary material Figs. 4(d) and 4(e)].

In addition to changes in EC size, quantitative analysis of VE-cadherin intensity on CCM1 LOF micro-vessels grown in distinctly sized HA suggested that HMW HA could have a beneficial impact on micro-vessel integrity [Figs. 4(c) and 4(d)]. Notably, our mRNA expression analysis predicted that LMW and HMW HA might be reduced in the ECM due to changes in HA synthesis and turnover when CCM1 is lost [Fig. 4(a)]; therefore, combined action of LMW and HMW HA in the ECM may create a CCM protective microenvironment.

**FIG. 5.**
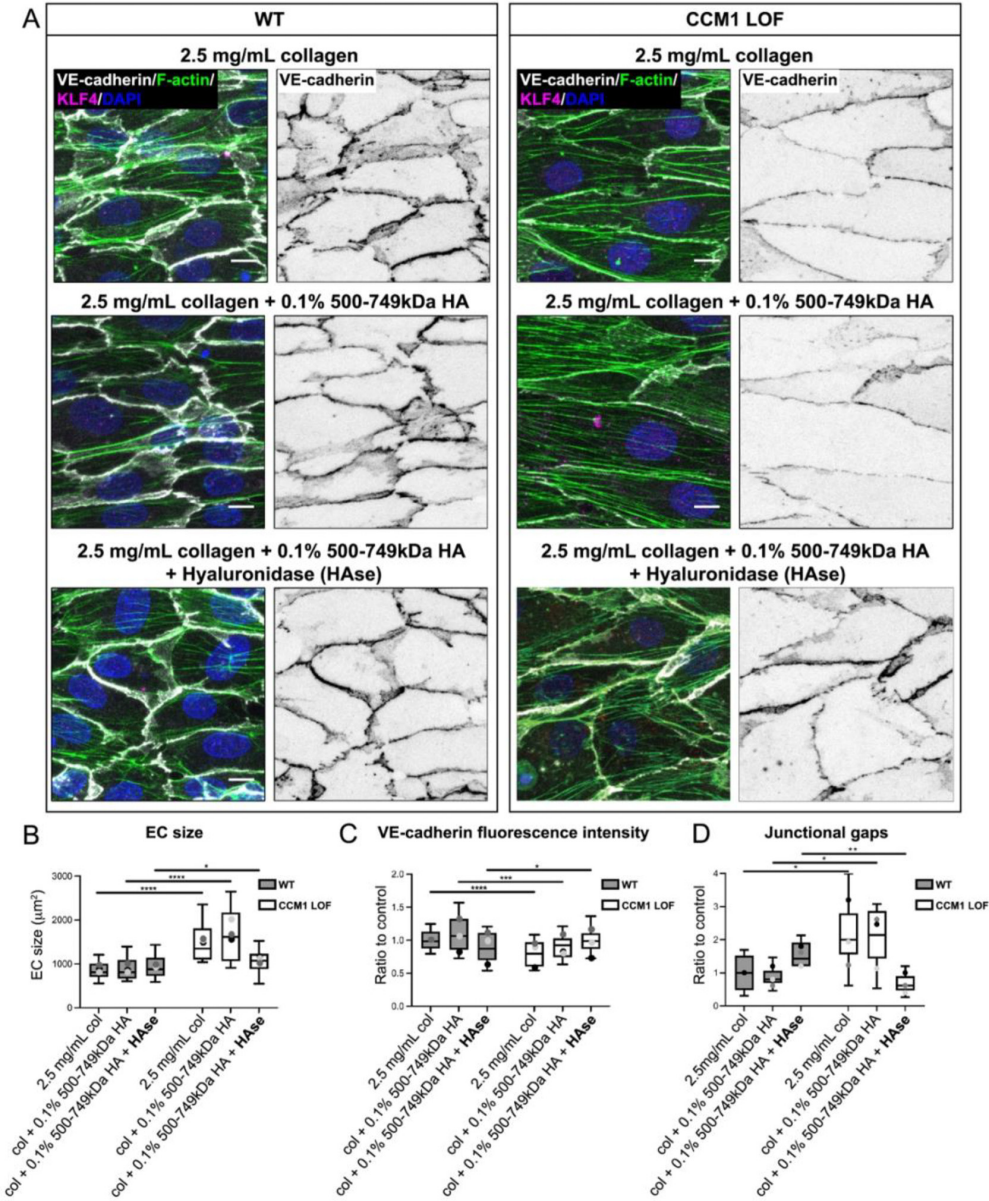
A combination of LMW and HMW HA in the ECM generates a CCM inhibitory environment. (a) Immunofluorescence of WT and CCM1 LOF ECs, seeded in 3D microfluidic devices. The composition of ECM is indicted for each condition. Cells were grown for 48 h post seeding and stained for VE-cadherin (white), Phalloidin (Actin) (green), KLF4 (magenta), and DAPI (blue). Scale bar: 10 *μ*m. (b) Quantification of WT and CCM1 LOF EC size, measured based on VE-cadherin staining. Box and whisker plot with mean value of each replicate represented as a dot with matching colors between WT and CCM1 LOF ECs. n = 3 replicates; 2.5 mg/ml collagen-I n = 46 WT and n = 33 CCM1 LOF ECs, collagen-I and 0.1% 500–749 kDa HA n = 52 WT and n = 38 CCM1 LOF ECs, collagen-I and 0.1% 500–749 kDa HA and HAse n = 38 WT and n = 47 CCM1 LOF ECs. Mann Whitney test ^****^p < 0.0001, ^*^p < 0.05, ns = no significant difference. (c) Quantification of VE-cadherin expression at cell–cell junctions in WT and CCM1 LOF ECs. Box and whisker plot with mean value of each replicate represented as a dot with matching colors between WT and CCM1 LOF ECs. n = 3 replicates; 2.5 mg/ml collagen-I n = 44 WT and n = 31 CCM1 LOF ECs, collagen-I and 0.1% 500–749 kDa HA n = 51 WT and n = 36 CCM1 LOF ECs, collagen-I and 0.1% 500–749 kDa HA and HAse n = 38 WT and n = 39 CCM1 LOF ECs. Student's t-test ^****^p < 0.0001, ^***^p < 0.001, ^*^p < 0.05. (d) Quantification of WT and CCM1 LOF junctional gaps. Box and whisker plot with mean value of each replicate represented as a dot with matching colors between WT and CCM1 LOF ECs. n = 3 replicates; n = 6 WT ROIs and n = 6 CCM1 LOF ROIs, collagen-I and 0.1% 500–749 kDa HA and HAse n = 6 WT and n = 6 CCM1 LOF ROIs, collagen-I and 0.1% <10 kDa HA, 0.1% 41–65 kDa HA and 0.1% 500–749 kDa HA n = 6 WT and n = 6 CCM1 LOF ROIs, Student's t-test ^****^p < 0.0001, ^**^p < 0.005, ^*^p < 0.05.

We investigated whether a mixture of HMW and LMW HA fragments would be the most favorable to recover EC size and micro-vessel integrity. To test this, we generated ECMs with a combination of LMW HA and HMW HA fragments by adding HAse to HMW HA hydrogels. Without altering KLF4 expression [supplementary material Figs. 5(a) and 5(b)], both EC size and VE-cadherin intensity were most improved in micro-vessels grown in the presence of LMW/HMW HA ECMs [Figs. 5(a)–5(c)]. As a more direct measure of vessel integrity, we quantified the number of junctional gaps and found that LMW/HMW HA ECMs significantly improved barrier integrity in CCM1 LOF micro-vessels, demonstrated by a reduction in junctional gaps [Fig. 5(d)]. ECM hydrogels composed of equal amounts of HMW HA, LMW HA and HA oligomers (<10 kDa) did not exert a similar rescue of EC size and junctional gaps [supplementary material Figs. 6(a)–6(c)]. These LMW/HMW composite ECMs were comparable in terms of stiffness [supplementary material Fig. 6(d)]. This implies that LMW HA provides the main protective cue and that it is essential that these LMW HA forms are present at proportionally higher levels than HMW HA.

**FIG. 6.**
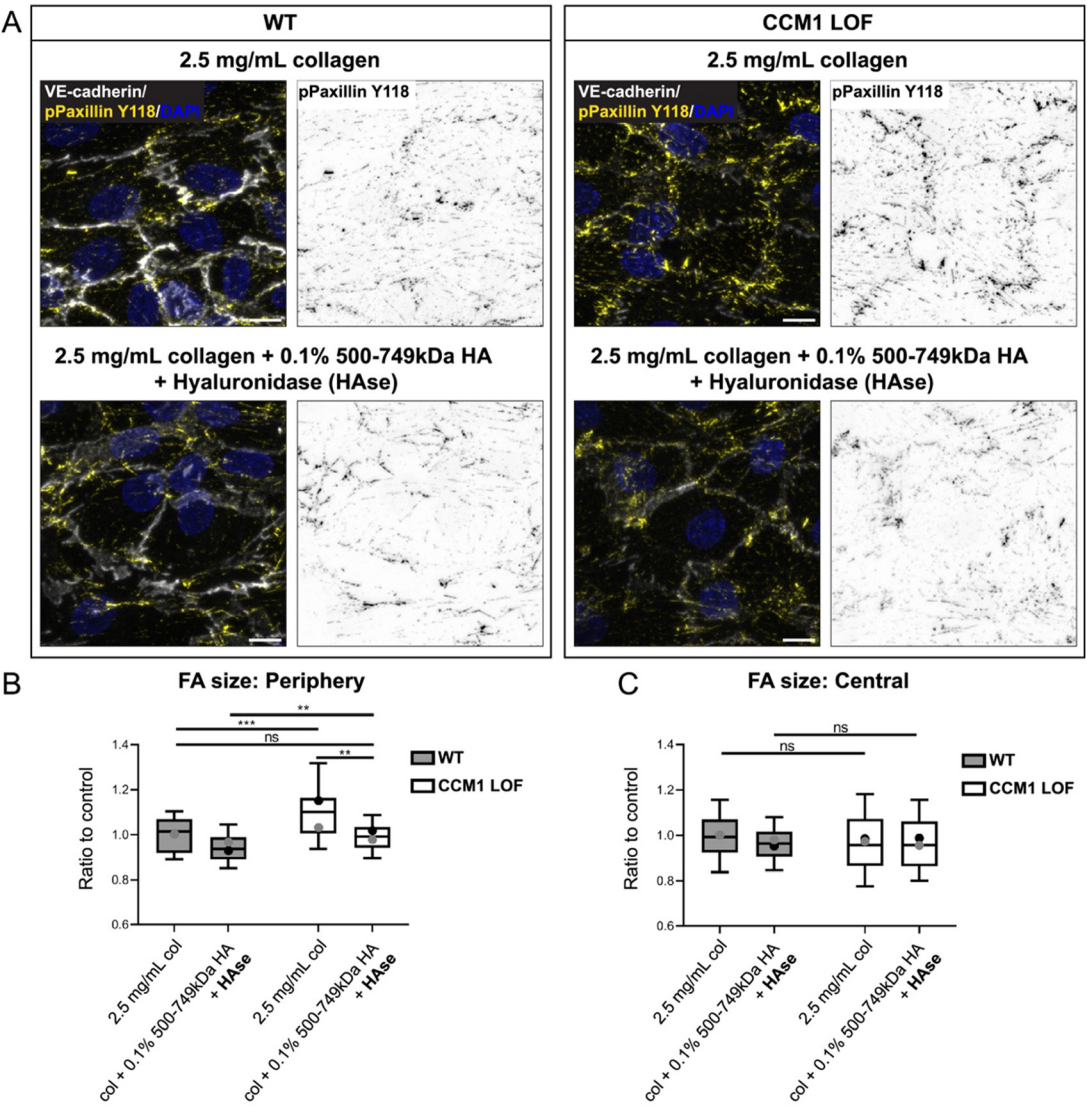
HA inhibits cell matrix adhesion signaling in CCM. (a) Immunofluorescence of WT and CCM1 LOF ECs, seeded in 3D microfluidic devices. The composition of ECM is indicted for each condition. Cells were grown for 48 h post seeding and stained for VE-cadherin (white), phosho-Paxilin (Y118) (yellow), and DAPI (blue). Scale bar: 10 *μ*m. (b) Quantification of WT and CCM1 LOF EC focal adhesion (FA) size at the periphery of each endothelial cell, measured based on pPaxillin staining. Box and whisker plot with mean value of each replicate represented as a dot with matching colors between WT and CCM1 LOF ECs. n = 2 replicates; 2.5 mg/ml collagen-I n = 660 WT and n = 399 CCM1 LOF ECs, collagen-I and 0.1% 500–749 kDa HA and HAse n = 612 WT and n = 597 CCM1 LOF ECs. Student's t-test, ^***^p < 0.001, ^**^p < 0.005. (c) Quantification of WT and CCM1 LOF EC focal adhesion (FA) size in the central region of each endothelial cell, measured based on pPaxillin staining. Box and whisker plot with mean value of each replicate represented as a dot with matching colors between WT and CCM1 LOF ECs. n = 2 replicates; 2.5 mg/ml collagen-I n = 660 WT and n = 399 CCM1 LOF ECs, collagen-I and 0.1% 500–749 kDa HA and HAse n = 612 WT and n = 597 CCM1 LOF ECs. Student's t-test, ns = no significant difference.

### LMW HA inhibits downstream activation of cell matrix adhesion signaling in CCM micro-vessels

Since loss of CCM1 has previously been shown to induce ectopic integrin signaling, leading to changes in EC shape, we explored whether the protective effect of LMW HA was due to changes in cell matrix adhesion. Both WT and CCM1 LOF micro-vessels were grown in 2.5 mg/ml collagen and LMW/HMW HA composites (2.5 mg/ml collagen + HMW HA of 500–749 kDa + hyaluronidase). Activated integrin receptors form multi-protein complexes that connect cells to the ECM, called focal adhesions (FAs). Integrin signaling activates intracellular tyrosine kinases of which FAK, in association with Src, can phosphorylate Paxillin at FAs.^53^ To interrogate FA abundance and distribution, we analyzed pPaxillin (phosphorylated at Y118) expression by immunofluorescence. In CCM1 LOF micro-vessels grown in 2.5 mg/ml collagen, we observed that pPaxillin positive FAs were particularly pronounced at the periphery of CCM1 LOF ECs [Fig. 6(a)]. Quantitative analysis confirmed that FAs located at the EC periphery were significantly larger [Fig. 6(b)], while in the center of the ECs, pPaxillin positive FAs were not significantly altered [Fig. 6(c)]. Interestingly, when CCM1 LOF micro-vessels grown in ECMs that were supplemented with HMW HA and Hyaluronidase, these FAs at the periphery were smaller in size and indistinguishable from those in WT micro-vessels grown in 2.5 mg/ml collagen [Figs. 6(a) and 6(b)]. Together, this data supports a model wherein LMW HA inhibits EC enlargement and junctional gap formation in CCM deficient vasculature by controlling cell–matrix adhesion signaling. Considering HA-based scaffolds are being developed as CNS therapeutics,^54,55^ further research into the mechanism of LMW HA in the CCM microenvironment might uncover new avenues to dampen CCM lesion growth.

## CONCLUSION

In light of accumulating evidence that non-cell autonomous signals play a crucial role in CCM lesion progression,^17,18,22,23^ we adapted a 3D micro-fluidic model^25,26^ to grow human CCM1 deficient micro-vessels. We validated that characteristic phenotypic changes such as EC enlargement, induction of KLF4, reduced VE-cadherin, and increased vessel permeability are all maintained when CCM1 LOF micro-vessels are cultured in a standardized ECM (2.5 mg/ml collagen).^13,15,56^ Reducing collagen content and, thus, ECM stiffness did not worsen CCM phenotypes, suggesting that restrictiveness of CCM lesions to CNS tissues is not solely explained by low tissue compliance. By transcriptional profiling, we identified that CCM1 LOF likely alters homeostasis of the main ECM component of the CNS, Hyaluronic acid (HA). When culturing CCM1 LOF micro-vessels in ECMs enriched with distinctly sized HA, we uncovered that changes in HA composition of the ECM can alter CCM phenotypes, independent of the key transcriptional inducer of CCMs, KLF4. In particular, supplementation with LMW HA could inhibit the increase in EC size that is characteristic to CCM deficient ECs. A combination of LMW/HMW HA in the ECM was most beneficial, with CCM1 LOF ECs not only retaining a normal cell size but also showing improved VE-cadherin coverage and importantly a significant decrease in junctional gaps, suggesting recovery of vessel integrity. HA has been reported to bind to Fibronectin,^57^ thereby reducing outside-in integrin signaling capacity.^58^ β1-Integrin overactivation has been associated with CCM.^23,24^ We validated that FAs are significantly enhanced in CCM1 LOF micro-vessels grown in 2.5 mg/ml collagen. We identified that this increase is spatially restricted with enlarged FAs found specifically at the EC periphery. The CCM inhibitory environment created by LMW/HMW composite ECMs reduced FAs at the EC periphery, supporting a mechanism whereby HA inhibits ectopic cell–matrix adhesion signaling in CCM ECs, leading to a normalization of EC size.

Together, this work underpins the relevance of HA homeostasis in CCM disease. With CCM lesions mainly found in the CNS, a HA rich tissue, CCM induced changes to the HA equilibrium are likely to be exacerbated and might play a role in the CNS restrictiveness of this disease.

Our findings further highlight the effectiveness of 3D micro-vessel modeling in examining the role of ECM components in CCM. This approach will be applicable to research aimed to investigate similar non-cell autonomous mechanisms in other vascular malformations and diseases, such as lymphatic malformations and aneurysms.

## METHODS

### Reagents

Primary antibodies: mouse α-VE-cadherin (WB 1:1000; sc-9989; Santa Cruz), rabbit α-KRIT1 (CCM1) (WB 1:1000; ab196025; Abcam), rabbit α-GAPDH (WB 1:5000; 2118; Cell Signaling), mouse α-VE-cadherin, conjugated to Alexa 647 (IF 1:250; 561567; Becton Dickinson), rabbit α-KLF4 (IF 1:250; 4038; Cell Signaling), rabbit α-phosphorylated Paxillin (Y118) (2D IF 1:1000; 3D IF 1:200; 69363; Cell Signaling), mouse α-phosphorylated Myosin Light Chain (S19) (IF 1:250; 3675; Cell Signaling), rabbit α-phosphorylated Myosin Light Chain (T18/S19) (IF 1:500; 3674; Cell Signaling), rabbit α-beta Catenin (IF 1:1000; C2206; Sigma Aldrich), Phalloidin, conjugated to Alexa 488 (IF 1:500; A12379; Thermo Fischer), and Phalloidin, conjugated to Alexa 647 (IF 1:500; ab176759; Abcam). For Western blotting, secondary antibodies, horseradish peroxidase (HRP)-conjugated goat α-mouse (Sigma Aldrich A4416; 1:10 000) and HRP-conjugated goat α-rabbit (Sigma Aldrich A0545; 1:10 000) were used. For immunofluorescence, Alexa Fluor 488 conjugated goat α-mouse (Thermo Fischer Scientific A-11001; 1:1000) and Alexa Fluor 568 conjugated goat α-rabbit (Thermo Fischer Scientific A-11036; 1:1000) were used. Additional reagents used: R&D Systems Culturex 3D Culture Matrix Rat collagen I (In vitro technologies; RDS344702001), Poly-L-Lysine (Sigma Aldrich; P8920), Dulbecco's Modified Eagle's Medium (Sigma Aldrich; D5648), PDMS (AIBN; 04019862), sodium hyaluronate (<10 kDa) (Lifecore Biomedical; HA5K-1), sodium hyaluronate (41–65 kDa) (Lifecore Biomedical; HA40K-1), sodium hyaluronate (500–700 kDa) (Lifecore Biomedical; HA700K-1), sodium hyaluronate (1–1.8 MDa) (Lifecore Biomedical; HA15M-1), and hyaluronidase from *Streptomyces hyalurolyticus* (Sigma Aldrich; H1136), this HAse acts on all types of HA, producing HA of smaller sizes.^59^

### Cell culture

Human umbilical vein endothelial cells (HUVECs) were purchased from LONZA (C2519A) and were cultured according to the supplier's recommendations, using EBM-2 basal medium (CC-3156), supplemented with EGM-2 SingleQuots supplements (CC-4176). Cells were trypsinized using Trypsin-EDTA (LONZA; CC-5012) and maintained for up to five passages. Human Embryonic Kidney (HEK)-293T cells were cultured in DMEM (Thermo Fischer Scientific; 11995073), containing 10% fetal bovine serum (Thermo Fischer Scientific; 10099141) and 100 U/ml penicillin and streptomycin (Thermo Fischer Scientific; 15070063). Cells were trypsinized, using Trypsin-EDTA (Thermo Fischer Scientific; 15400054). All cell lines were cultured in a humidified 37 °C incubator with 5% CO_2_.

### Lentivirus production

The guide (g)RNA sequence targeting *Homo sapiens KRIT1* (5′-GTATTCCCGAGAATTGAGACTGG-3′) was selected based on the Zhang lab online gRNA prediction tool (http://crispr.mit.edu/). The gRNA was subsequently cloned in lentiCRISPRv2 vector (Addgene; 52961), flanked by Esp3I (New England Biolabs; R0734) restriction sites. Lentiviral particles were produced in HEK 293T cells, which were co-transfected with the gRNA containing lentiCRISPRv2 vector together with the packaging vectors pMDLg/pRRE (Addgene; 12251), pRSV-Rev (Addgene; 12253), and pMD2.G (Addgene; 12259) using polyethyleneimine (Sigma Aldrich; 764604) as a transfection reagent. Supernatants containing viral particles were collected 48 and 72 h post transfection, concentrated with Lenti-X concentrator (Clontech; 631232) and used to infect HUVECs. Transduced HUVECs were selected with 1 *μ*g/ml Puromycin (Sigma Aldrich; P8833).

### Generating micro-vessels and ECM hydrogels

The microfluidic devices were made as previously described,^25,26^ using 2.5 mg/ml collagen-I (Rat tail, In vitro technologies; RDS344702001) as a standard extracellular matrix. HUVECs were close to confluency on plastic plates prior to seeding into the ECM and were maintained on a rocker in a humidified 37 °C incubator with 5%CO_2_ to form tight tubes. When indicated, HA polysaccharides (Lifecore Biomedical) were pre-mixed with collagen-I in the hydrogels to facilitate the formation of an interconnected^50,51^ and uniform ECM. Hyaluronidase from *Streptomyces hyalurolyticus* (Sigma Aldrich; H1136) was also pre-mixed into the matrix at 5 U/ml.

### Immunofluorescence and imaging

Cells, grown on glass cover slips were fixed in 4% formaldehyde solution in PBS for 10 min. Permeabilization was done, using 0.3% Triton X100 in PBS. Cells were blocked in a blocking buffer, consisting of 10% goat serum (Sigma Aldrich; G6767) in PBS for 1 h at room temperature. Primary and secondary antibodies were diluted in blocking buffer in the above indicated dilutions and incubated for 1 h each with 6 washing steps with PBS for 10 min in between. Final washing steps were carried out with the addition of 4′,6-diamidino-2-phenylindole. Cover slips were mounted on microscope slides using Mowiol. Fluorescent still images were acquired at a Zeiss AxioImage M2 upright microscope, equipped with a 63× N.A. 1.4 Oil objective and an Axiocam 506. Single plane confocal images were acquired using a Zeiss Axiovert 200 Inverted microscope with an LSM 880 confocal scanner, equipped with a 63× N.A. 1.4 Oil objective. collagen fibers were imaged on a Leica DMi8 inverted microscope with a SP8 Galvo and resonant confocal scanner, equipped with a 40× N.A. 1.1 Water objective. Microfluidic devices were fixed sequentially in 1% formaldehyde in PBS, containing 0.05% Triton X100, for 90 s, followed immediately by 4% formaldehyde in PBS for 15 min at 37 °C on a rocking platform. Tubes were permeabilized with 0.5% Triton for 10 min at 37 °C and blocked for 4 h in 10% goat serum in PBS at 4 °C prior to staining or using 2% bovine serum albumin in PBS supplemented with 10% fetal bovine serum at 4 °C on a rocking platform overnight for pPaxillin immunofluorescence. Primary and secondary antibodies were diluted in blocking buffer in the above indicated dilutions and incubated overnight at 4 °C, with an overnight PBS wash between primary and secondary incubations. Final washing steps were carried out with the addition of 4′,6-diamidino-2-phenylindole for 5 min, before washing with PBS for 30 min. Z-stack image acquisition was performed on a confocal laser scanning microscope (Zeiss Axiovert 200 inverted microscope with LSM 710 Meta Confocal Scanner) using 40× NA 1.1 water immersion objective and 1 *μ*m Z step size.

### Vessel permeability assay

Luminicell Tracker Vascular 670 nanoparticles (Luminicell Pte Ltd) were diluted at 1:50 in pre-heated EGM2 and added to the media ports of each micro-vessel. Devices were incubated for 20 min on a rocking platform at 37 °C. Micro-vessels were subsequently washed once with PBS followed by fixation and immunofluorescence as described above.

### Western blotting

Cell pellets were lysed in a lysis buffer containing 50 mM Tris (pH 8.0), 150 mM NaC, 1% Triton-X100, 10% glycerol and protease inhibitors cocktail (Sigma #04693116001). Protein concentration was measured using the Bradford method,^60^ prior to running the Western blots.^61^ Blocking was done in a 3% BSA solution in PBS, containing 1 mM EDTA and 0.05% Tween-20. Primary and secondary antibodies were diluted in the blocking solution and used according to the indicated concentrations with washing steps done in Tris-based saline with Tween 20 (TBST). Signals, emitted from the horseradish peroxidase were visualized by enhanced chemiluminescence (ECL) (BioRad #1705060) and imaged with Chemidoc (BioRad).

### Rheology

Rat tail collagen gels (In vitro technologies; RDS344702001) of different concentrations (1.25–2.5 mg/ml, pH 8–9, DMEM) and composites of collagen (2.5 mg/ml) and hyaluronan (1–10 mg/ml) of different molecular weights (<10 kDa–1.8 MDa; LifeCore Biomedical) were analyzed using an Anton Paar MCR-502 WESP rheometer. Samples were gelated *in situ* between plates by transferring the samples to a pre-cooled (5 °C) bottom stainless-steel plate (Anton Paar) using setting a gap of 0.5 mm with a stainless-steel parallel plate PP-25 (25 mm diameter, Anton Paar). Samples were trimmed and a thin layer of silica oil was placed around the sample. Gelation was induced with a temperature ramp from 5 to 37 °C (1 °C min^−1^) and further incubated for 2 h at 37 °C. Changes in the viscoelastic properties of the sample [G′ and G″ (Pa)] were monitored by applying oscillatory shear at a constant strain of 0.5% and frequency of 1 Hz.

### Quantitative RT-PCR

Cells were harvested directly in Buffer RLT with β-mercaptoethanol and RNA processed directly using the RNeasy Mini Kit (Qiagen), with on-column DNase digestion according to the manufacturer's instructions. RNA concentration was measured using a NanoDrop spectrophotometer and an equal starting concentration of RNA was for each sample was used for reverse transcription. Reverse transcription was performed using Superscript III Reverse Transcriptase (ThermoFisher) with Oligo dT priming. Primer sequences are detailed in Table I. Quantitative PCR was performed using SYBR green reagent (Applied Biosystems) on a QuantStudio 7 Flex Real-Time PCR System (ThermoFisher) in 384 well plates (Applied Biosystems) and relative gene expression was determined using the change-in-threshold (2^-DDCT^) method, using Hypoxanthine Phosphoribosyltransferase 1 (HPRT) as a housekeeping control.

**TABLE I.** qPCR primers sequences.

Gene	Primer 1	Primer 2
*KLF4*	CGTTGACTTTGGGGTTCAGG	GCGAACGTGGAGAAAGATGG
*HAS1*	GCTCGGAGATTCGGTGGAC	CACGTCCCCACCAACAGC
*HAS2*	GCAAAAATGGGGTGGAAAAAG	TGGGTCAAGCATAGTGTCTGAATC
*HAS3*	TCCAGGTGTGCGACTCTGAC	CGCTGCTCAGGAAGGAAATC
*HYAL1*	AGGGCACAGGGAAGTCACAG	TCATCCAGGGGCAGAAAGTG
*HYAL2*	CACGGGGCTTAGTGAGATGG	GGTCTCCGTGCTTGTGGTGT
*HYAL3*	TATGTCCGCCTCAGACACCG	TGCCAGCACTCCTCCTCAGA
*HYAL4*	AGGAGAAAGTGCTGCCTTGGG	GCAGCTCTGGTCACATTGGCT
*CEMIP*	TTCAACAAGGGCGACTGGAT	TGCTCCACTTTGTCCATCTGC
*CEMIP2*	TCCAGCACGGGGTTACTGTT	TGTGGCTGCTTGGATCTTGA
*SPAM1*	TGTTGCTCTGGGTGCTTCTG	CATTTTGGCTGCTAGTGTGACG
*HPRT*	TCAGGCAGTATAATCCAAAGATGGT	AGTCTGGCTTATATCCAACACTTCG

### Image processing and quantification

All image processing and analysis was performed using Fiji, Image J (National Institutes of Health).

#### Nuclear KLF4 expression quantification

By using the DAPI staining as a reference, the nuclei were selected using the free-hand tool (FIJI). KLF4 fluorescence intensity was measured within these nuclear regions of interest (ROIs).

#### Vessel diameter quantification

Of each micro-vessel, three brightfield images were captured along the length of the vessel. On each image, five evenly spaced points across the length of each image were measured with the straight-line tool (FIJI). The mean was calculated by averaging all measurements for each vessel and reported as vessel diameter.

#### VE-cadherin fluorescence intensity quantification

Using an automated Fiji script, a band of 4 *μ*m in size was drawn around the cell periphery and VE-cadherin intensity was calculated within these junctional ROIs.

#### Focal adhesion size quantification

Images were processed through the cell pose cell analyzer pipeline, using the cyto2 model for cell segmentation. The cell pose mask outputs were further processed in ImageJ, removing any cells which were incomplete, and ROIs were adjusted manually if Cell Pose segmentation was not accurate. The pPaxillin channel was threshold using default dark before being converted to a binary mask. The focal adhesion area was measured within each cell using particle analyzer (size: 0.25 *μ*m^2^—infinity).

### Statistical analysis

We performed all statistical analysis using Prism 9 (GraphPad). All quantifications were based on data acquired from at least two replicates. D'Agostino-Pearson test was applied to test normal distribution of the data points. When the data were normally distributed a Student's t-test was used for comparison of two means. When the data did not follow a normal distribution, a Mann–Whitney test was used for comparison of two means. The threshold for significance was taken as p < 0.05.

## SUPPLEMENTARY MATERIAL

See the supplementary material for Fig. 1: induced pMLC expression and F-actin stress fibers in 2D cultured CCM1 LOF ECs; Fig. 2: mechanical and structural properties of collagen hydrogels and collagen-HA composites; Fig. 3: distinct HA environments do not induce changes in ectopic KLF4 expression; Fig. 4: 41–65 kDa HA is required and sufficient to reduce CCM1 LOF EC size; Fig. 5: KLF4 expression analysis in HMW HA and LMW/HMW HA composite ECMs; Fig. 6: collagen-HA ECMs comprised of HMW, LMW and HA oligomers at equimolar ratios do not rescue CCM phenotypes.

## Data Availability

The data that support the findings of this study are available from the corresponding author upon reasonable request.
